# Rhesus Monkey Rhadinovirus Isolated from Hemangioma Tissue

**DOI:** 10.1128/MRA.01347-19

**Published:** 2020-03-19

**Authors:** Armin Ensser, Koji Yasuda, William Lauer, Ronald C. Desrosiers, Alexander S. Hahn

**Affiliations:** a Institute of Clinical and Molecular Virology, Friedrich-Alexander University (FAU) Erlangen-Nürnberg, Erlangen, Germany; b New England Primate Research Center, Southborough, Massachusetts, USA; c Department of Pathology, Miller School of Medicine, University of Miami, Miami, Florida, USA; d Junior Research Group Herpesviruses, German Primate Center–Leibniz Institute for Primate Research, Göttingen, Germany; KU Leuven

## Abstract

We isolated a rhesus monkey rhadinovirus, isolate RRVmmu 209-07, from hemangioma tissue. The virion DNA was sequenced by Illumina-based sequencing. Isolate RRVmmu 209-07 is highly similar overall to RRV 26-95, but considerable differences exist in the 3′ region of the genome.

## ANNOUNCEMENT

A male rhesus macaque (animal 209-07) experimentally infected with simian-human immunodeficiency virus for 559 days at the New England Regional Primate Research Center (1) presented at study endpoint with hemangiomas in the mandibular gingiva, lower abdomen, and scrotum.

Hemangioma tissue samples were passed through a 100-μm cell strainer and added to primary rhesus monkey fibroblasts. As cytopathic effect (CPE) became visible, 11 ml of the cell culture supernatant was cleared by low-speed centrifugation and pelleted at 26,892 × *g* for 2 h, and the supernatant was removed except approximately 1 ml, in which the pellet was reconstituted. Encapsidated DNA was isolated as described previously (2) by DNase digest followed by heat inactivation, proteinase K digest, phenol-chloroform extraction with one extra phenol extraction, and isopropanol precipitation. The DNA was resuspended in 40 μl water and 2 μl Tris-EDTA (TE) buffer.

DNase-protected DNA was used to prepare a double-indexed Illumina-compatible library using the Nextera DNA sample preparation system (Illumina, San Diego, USA). In two runs, 384,400 paired reads of 150 nucleotides and 1,067,294 paired reads of 250 and 50 nucleotides were obtained on the Illumina MiSeq system using the MiSeq reagent kit (300 cycle). Illumina reads were quality trimmed, *de novo* assembled, and analyzed using CLC Genomics Workbench 9.5 and 11 (Qiagen Aarhus S/A, Denmark) at default settings. A BLAST (3) search with individual contigs demonstrated the presence of a rhesus monkey rhadinovirus (RRV; order *Herpesvirales*, family *Herpesviridae*, subfamily *Gammaherpesvirinae*). Quality-trimmed reads were aligned to the RRV reference genome (strain 17577; GenBank accession number NC_003401) (4). A consensus sequence was derived by assembly of the original reads to the reference; a new consensus was reiteratively derived, used for another round of assembly (821,931 aligned reads; average coverage, 619×), and annotated with the conserved open reading frames (ORFs) of RRV. The final sequence, after trimming of repetitive heavy DNA (H-DNA), consists of 129,945 nucleotides with a GC content of 52%, representing the complete light DNA (L-DNA).

BLAST analysis (3) of isolate RRVmmu 209-07, at default settings, indicated 99.71% identity (where sequences aligned) to isolate RRV 26-95 (accession number AF210726) (5) at 98% coverage (percentage of sequence that was aligned) and 99.10% identity to isolate 17577 at 98% coverage. In *orf22* and *orf47*, which define the sequence subgroups of RRV 26-95 and 17577, respectively (6), our isolate was 99% and 100% identical to RRV 26-95 on the amino acid level. Small differences exist in comparison to RRV 26-95 and to 17577 in the microRNA cluster and are more pronounced in the 3′-end genomic region, where the homolog of the Kaposi’s sarcoma-associated herpesvirus (KSHV) K15 gene should be located (7). In the region between *orf75* and the 3′ end of the genome, the identity of RRVmmu 209-07 to RRV 17577 is at 79.88%, lower than its identity to RRV 26-95 at 85.05%, where sequences were aligned by BLAST. Several indels and base substitutions clearly discriminate the sequences of RRVs 17577 and 26-95 in this locus. Whether viral gene products were expressed in the hemangioma tissue remains unclear, as further studies were hampered by a lack of well-preserved tissue specimens. Overall, our findings indicate a thus far underappreciated diversity in the 3′-end genomic region of RRV.

### Data availability.

The sequence of RRVmmu 209-07 has been deposited in GenBank (accession number MN488836). Sequence read data are available under BioProject number PRJNA594832 and BioSample number SAMN13531421.
